# Psychedelic therapy in the treatment of addiction: the past, present and future

**DOI:** 10.3389/fpsyt.2023.1183740

**Published:** 2023-06-12

**Authors:** Rayyan Zafar, Maxim Siegel, Rebecca Harding, Tommaso Barba, Claudio Agnorelli, Shayam Suseelan, Leor Roseman, Matthew Wall, David John Nutt, David Erritzoe

**Affiliations:** ^1^Centre for Psychedelic Research, Department of Brain Sciences, Faculty of Medicine, Imperial College London, London, United Kingdom; ^2^Neuropsychopharmacology Unit, Department of Brain Sciences, Faculty of Medicine, Imperial College London, London, United Kingdom; ^3^Clinical Psychopharmacology Unit, University College London, London, United Kingdom; ^4^Invicro, London, United Kingdom

**Keywords:** psychedelic therapy, addiction, neuropsychopharmacology, fMRI, PET, randomized controlled trial (RCT), real world evidence (RWE), drug developement

## Abstract

Psychedelic therapy has witnessed a resurgence in interest in the last decade from the scientific and medical communities with evidence now building for its safety and efficacy in treating a range of psychiatric disorders including addiction. In this review we will chart the research investigating the role of these interventions in individuals with addiction beginning with an overview of the current socioeconomic impact of addiction, treatment options, and outcomes. We will start by examining historical studies from the first psychedelic research era of the mid-late 1900s, followed by an overview of the available real-world evidence gathered from naturalistic, observational, and survey-based studies. We will then cover modern-day clinical trials of psychedelic therapies in addiction from first-in-human to phase II clinical trials. Finally, we will provide an overview of the different translational human neuropsychopharmacology techniques, including functional magnetic resonance imaging (fMRI) and positron emission tomography (PET), that can be applied to foster a mechanistic understanding of therapeutic mechanisms. A more granular understanding of the treatment effects of psychedelics will facilitate the optimisation of the psychedelic therapy drug development landscape, and ultimately improve patient outcomes.

## Introduction

Addiction is a chronic relapsing medical condition with a global prevalence for which there are very limited effective treatment options. Recent estimates predict around 164 million individuals globally are currently suffering from addiction (1). In the western hemisphere alone, it is estimated that 5–6% of individuals suffer from a substance-related issue, and in recent years there has been accumulating concern over the prevalence of behavioral addictions. For instance, the World Health Organisation (WHO) has estimated that up to 6% of young adults reach the threshold for problem gambling (2). Notably, the global prevalence of all substance use disorders (SUDs) has increased substantially between 1990 and 2016, with alcohol dependence being the most prevalent (3). Furthermore, there has been a consistent increase in drug-related deaths since the turn of the millennium, with 2017 seeing 35% higher deaths compared to 2000, and with worrying trends in recent years from overdose with opiates and synthetic drugs seen most acutely in the US, Scotland, and England (3). This trend is seen globally with the WHO reporting increases in drug-related deaths in every continent of the world (4). Addiction is an economically crippling disorder exacting more than $442 billion annually in economic burden (5). This has far-reaching deleterious consequences that go beyond the individual, impacting employment, productivity, public health and the judicial-legal system.

Despite the morbidity of SUDs and behavioral addictions, fewer than 10% of the 22 million Americans identified as needing treatment are able to access specialist services (6, 7). In the UK, only 9000 individuals were in contact with specialist gambling treatment clinics (6) in 2019–2020, despite a recent YouGov poll estimating that up to 1.4 million individuals living in the UK reach the threshold for problematic gambling (8). These figures lay bare the inadequate provision of support and access to treatment for addictions. The reasons for a lack of access to adequate services are multifactorial and are reported eminently in the UK government-commissioned independent review of drugs from August 2021 by Dame Carol Black (9). Her report furthers the calls from NIDA’s 2019 medication development priorities paper, highlighting the pressing socio-economic need for the development of more effective treatments through innovative and mechanistic scientific programs to improve translation to the clinic (10).

The current clinical treatment paradigm for addictions is a psychosocial intervention with adjunctive pharmacotherapy. These current treatments and interventions for addictions provide limited success with 20% of individuals relapsing within 1 month and a further 40% within 6 months (11). The effectiveness of treatments for alcohol addiction is among the lowest of all mental health disorders, with only 3 licensed pharmacotherapies available and only 9% of individuals with this disorder receiving such treatments (12). The situation is arguably worse for individuals with other substance or behavioral addictions, which have fewer or no clinically efficacious medications available (11).

Despite the limited current availability of effective treatments, there has been a growing body of evidence since the mid-20th century indicating the therapeutic effects of psychedelics in treating addiction including, the ‘classic psychedelics’; lysergic acid diethylamide (LSD), psilocybin, dimethyltryptamine (DMT) ayahuasca (a drink that contains DMT and a monoamine oxidase inhibitor that prevents its breakdown in the gut), 5-Methoxy-N,N-dimethyltryptamine (5-MeO-DMT, from the Sonora dessert toad), mescaline (from the peyote cactus) all of which act as agonists of the serotonin 2A receptor (5-HT_2A_R). In addition, there are the ‘non-classic psychedelics’; ketamine, 3,4-methylenedioxymethamphetamine (MDMA) and ibogaine (from the Iboga plant).

This research was catalyzed in the middle of the 20th century with evidence coming from case studies, observational and naturalistic research, and more recently modern-era double-blind randomized controlled trials (RCTs). The last decade has seen an expansion into understanding further the mechanism of action of these compounds, using modern biomedical techniques spanning from preclinical molecular biology to *in vivo* human neuroimaging. This review aims to chart the current evidence for psychedelic therapy, including both classic and non-classic psychedelics, in the treatment of addiction and summarize the current state of knowledge on the mechanisms of action of these compounds. We will finish this review by highlighting several research avenues that could profitably be explored over the coming years to optimize the development of psychedelic therapy for this indication.

### Historical studies of psychedelic therapy for addiction: 1934–2000

One of the first documented accounts in Western medicine was in 1934 when Bill Wilson, the co-founder of alcoholics anonymous, embarked on his fourth attempt at recovery from his own alcoholism with the aid of an experimental treatment: an admixture containing henbane and belladonna; plants that contain tropane alkaloids that have psychedelic-like effects. It was under the influence of this medicine that Wilson reported an experience of a ‘bright white light’ and ‘a feeling of great peace’ which he interpreted as a spiritual and self-transcendent awakening. Following this treatment, he never drank alcohol again and went on to develop the international mutual aid fellowship Alcoholics Anonymous. Later having tried LSD he famously said, “If, therefore, under LSD we can have a temporary reduction, so that we can better see what we are and where we are going — well, that might be of some help. The goal might become clearer. So, I consider LSD to be of some value to some people, and practically no damage to anyone.” Bill Wilson, founder of Alcoholics Anonymous (13).

### Classic psychedelics

Over 1000 papers were published during the mid-late twentieth century describing the treatment of 40,000 patients with a psychiatric disorder, including addiction, with classic psychedelics, especially LSD (14). Studies relevant to addiction are summarized in the following sections.

### LSD

Lysergic acid diethylamide (LSD) was first synthesized in 1938, though its psychological properties were not discovered until 1943 (15). LSD binds to a range of neuroreceptors including the 5-HT_2A_R which pharmacologically defines the mechanism of action of classic psychedelics [See review; (16)]. LSD is currently being investigated in clinical trials for several neuropsychiatric disorders (17).

Wilsons’s inaugural judgments of the clinical potential of psychedelic compounds and later LSD (18) instigated substantial research of this drug in the treatment of alcohol dependence in the 1950 and 1960s (19). This research culminated in a meta-analysis published in 2012 (20) of 6 randomized controlled trials of 536 participants treated with one dose of LSD for their alcoholism, showing a significant beneficial effect of LSD on reducing alcohol misuse when compared with placebo [Odds ratio (OR), 1.96; 95% CI, 1.36–2.84; *p* = 0.0003]. This OR far surpasses all current therapies for alcoholism with naltrexone having a modest OR of 0.69 (95% CI, 0.41–1.18; *p* = 0.17) when compared with placebo. In the LSD group, 59% of individuals improved versus 38% in the placebo arm at the first follow-up. There were significant reductions in alcohol misuse seen at both three and 6 months follow-ups, but this effect was not significant at 12 months. For complete alcohol abstinence, there was an OR of 1.80 (CI 1.07–3.04) in the LSD condition at 3 months (20). Given these analyses, the number needed to treat for LSD for alcoholism comes out at six, whereas for acamprosate it is 12 and for nalmefene 20 (21).

Also in the early 1970s, research with LSD was being conducted on patients with heroin addiction. Seventy-eight inmates from a correctional institution were randomly assigned to either: one-dose of LSD-assisted psychotherapy (300–350 micrograms) or an outpatient clinic program with weekly group psychotherapy as a control. There were a total of 37 completers in each group and at both the 0–6 months and 7–12 months follow-ups, the LSD group had significantly higher rates of abstinence than controls. At 12 months only 5% of the control but 33% of the LSD group had confirmed abstinence (22). There were important differences between the groups apart from the LSD administration, such as 6 weeks of inpatient care for the LSD group and not the control group, which could confound the outcomes. The scale of these results, which were biologically confirmed through urinalysis at a 12-month follow-up, is impressive as a current-day study found that 91% of patients receiving standard treatment for opioid dependence relapsed at 6 weeks follow-up (23). A poignant quote from one of the participant’s (Leonard N.) experiences is described in the paper: *‘The two experiences of heroin and LSD are like night and day. Heroin is night, a time to sleep and with sleep, nothing comes but a dream. But with LSD, it is like dawn, a new awakening, it expands your mind, it gives you a brand-new outlook on life’* (22).

### Non-classic psychedelics

#### Ketamine

Ketamine is a dissociative anesthetic used both for medicinal and recreational purposes. Ketamine is an NMDA receptor antagonist and is thus classified as a non-classic psychedelic. Used for surgical anesthesia since the 1970s, it has since shown promising results for pain relief [for review see (24)], treatment-resistant depression [for review see (25)], and addiction [see review by Ivan Ezquerra-Romano et al. (26)]. In the 1990s the first study of the use of ketamine combined with therapy in the treatment of alcohol addiction took place in Russia. The study was non-randomized and allowed patients to choose between ketamine-psychedelic therapy (KPT) or conventional treatment. It was found that in the patients given three doses of 2.5 mg/kg (intramuscular) ketamine combined with psychodynamic psychotherapy 66% (versus 24% in the control group) achieved abstinence over 12 months follow-up (27).

These data were supplemented with an analysis of the psychological mechanisms of KPT, which reported harmonization of personality traits, emotional attitudes to self and others, positive changes in life and purpose and an increase in insight and spirituality (27). Further, neurobiological effects were assessed using electroencephalography (EEG). They found increased theta-wave activity power? in cerebrocortical brain regions, which they claimed as evidence for the reinforcement of reciprocal limbic-cortex interactions during the KPT session, and speaks to a potential neuropharmacological therapeutic mechanism of restoring dysfunctional corticolimbic activity.

#### Ibogaine

Ibogaine is a psychoactive alkaloid derived from the roots of a plant native to Gabon and central Africa called Tabernanthe iboga. Ibogaine binds to numerous neuroreceptors (28) though its primary mechanism of action is not mediated through the 5-HT_2A_R but through interaction with multiple neuroreceptor systems. Ibogaine has been used in traditional African shamanic practices for centuries and it induces a state of ‘oneirism’ or wakeful dreaming (29). Participants often report visual hallucinations and flashbacks of major prior life events with effects lasting up to 3 days (29). An open-label case series of 33 patients with opioid addiction who were treated between 1962 and 1993 and dosed with 19 (±7) mg/kg found that 25 of the patients showed resolution of signs of opioid withdrawal without further drug-seeking until the end of post-treatment observation at 72 h. In the 1990’s the US National Institute of Drug Abuse (NIDA) funded a phase 1 study into the effects of Ibogaine for opiate withdrawal. The study was halted part way through for cardiac safety concerns but the individuals who went through the study showed no sign of opiate withdrawal (30). To date, there has been no RCT evidence for Ibogaine, although efforts are now underway to restart this clinical development pipeline.

### Present day (2000–2023): real-world evidence of psychedelic therapy for addiction

For more than 50 years, prohibition effectively ceased clinical research into psychedelic compounds as a result of their placement in schedule 1 of the 1971 convention on psychotropic substances by the United Nations (UNODC, 1971). This ban states that these drugs have “no evidence of medical value” (UNODC, 1971) and has heavily impacted “an otherwise promising development of a novel treatment paradigm in mental health” (31). Despite these restrictions, the collection of real-world evidence for the therapeutic use of psychedelics has continued, in the form of retrospective, observational and naturalistic studies. Importantly, such real-world evidence can complement lab-based RCTs by indicating an intervention’s effectiveness in a more ecologically valid fashion and with more external validity than formal clinical trials can provide (32). Further, this data allows for the assessment of patients with multiple morbidities, with doses tailored to their clinical needs, which subsequently provide researchers with novel insights into parameters to design future studies (32). Below we summarize the evidence from such studies in individuals with addiction using classic and non-classic psychedelics for therapeutic purposes and have summarized these findings in Table 1.

**Table 1:** Observational studies of classic and non-classic psychedelic in addiction.

Publication	Link	Type of study	Addiction	Depend status	Measures used to measure addiction characheristics (if repeated = scale used both as screening and during the study)	Psychedelic drug	Dose	Sample size	Results	Time since relevant psychedelic experience	Statistically sign. Associations with acute measures
Alper 1999	https://pubmed.ncbi.nlm.nih.gov/10506904/	Open label case series	Opioid use disorder	DSM IV	Non specified, clinical assessment	Ibogaine	19.3 ± 6.9 mg/kg	33 (treated between 1962 and 1993)	25 patients showed resolution of the signs of opioid withdrawal without further drug seeking within 24 h and this was sustained until the end of post-treatment observation (72 h)	/	/
Mash 2001	https://pubmed.ncbi.nlm.nih.gov/11705106/	Observational study	Opioid use disorder	DSM IV	Opiate-Symptom Checklist (OP SCL), objective opioid withdrawal scale (OOWS)	Ibogaine	10 mg/kg	32	OOWS and OP-SCL scores significantly decreased 12, 24, and 36 h post-ibogaine treatment (*p* < 0.05)	/	/
Doering-Silveira 2005	https://repositorio.unesp.br/bitstream/handle/11449/159021/WOS000381098900007.pdf?sequence=1	Cross selectional study (adolescent ayahuasca users vs. national normative sample)	General drug consumption (no addiction)	/	WHO criteria for drug use	Ayahuasca	/	23 aya users vs. 23 controls	Significantly lower last-year and last-month alcohol consumption (p < 0.05)	/	/
Fabregas 2010	https://www.sciencedirect.com/science/article/abs/pii/S0376871610001699?via%3Dihub	Cross selectional study (ayahuasca users vs. national normative sample)	Polidrug use	Addiction Severity Index (ASI)	Addiction Severity Index (ASI), Questions about past and current drug use	Ayahuasca	/	127 ayahuasca users vs. 115 controls	Significantly lower scores in ASI subscales and current drug use (except for cannabis) in aya consumers (p < 0.05)	/	Possible role of church and community mentioned as an adjunctive explanation of the effects
Shenberg 2014	https://pubmed.ncbi.nlm.nih.gov/25271214/	Retrospective observational study	Polidrug abusers (alcohol, cannabis, cocaine and crack; no opioids exept in one patient)	DSM IV	No validated questionnaire, clinical assessment performed via interviews	Ibogaine	17 mg/kg (single/multiple treatments tailored to individual needs)	75	Participants treated once reported abstinence for a median of 5.5 months (p < 0.001) and those treated multiple times for a median of 8.4 months (*p* < 0.001)	/	/
Noller 2017	https://www.tandfonline.com/doi/full/10.1080/00952990.2017.1310218	Observational study	Opioid use disorder	DSM IV	Addiction Severity Index-Lite (ASI-Lite), objective opioid withdrawal scale (OOWS)	Ibogaine	31.4 ± 7.6 mg/kg	14	ASI-lite scores significantly decreased from baseline to 12-months follow up (p < 0.002), SOWS scores significantly decreased from 25.21 ± 12.57 pre-ibogaine to 14.21 ± 14.08 at 12–24 h post-ibogaine (*p* = 0.015)	/	/
Pisano 2017	https://pubmed.ncbi.nlm.nih.gov/28196428/	Association study	Opioid use disorder	NSDUH data from 2008 to 2013, subpopulation of individuals 18 or older who had used illicit opioids (NSDUH’s 10 dependence criteria).	/	LSd, Psilocybin, mescaline, peyote, DMT	/	Respondents with a history of illicit opioid use (44000)	Psychedelic use is associated with 27% reduced risk of past year opioid dependence (weighted RR = 0.73 (0.60–0.89) *p* = 0.002)	/	/
Thomas 2013	https://pubmed.ncbi.nlm.nih.gov/23627784/	Observational prospective study	Tobacco	4 Week Substance Use Scale (4WSUS)	/	Ayahuasca ceremonies focused on addiction	/	12 (polydrug users)	Decreases (non sign)		Yes
			Cannabis	4 Week Substance Use Scale (4WSUS)	/	Ayahuasca ceremonies focused on addiction	/		No effect		
			Alcohol	4 Week Substance Use Scale (4WSUS)	/	Ayahuasca ceremonies focused on addiction	/		Decreases (non sign)		Yes
			Opioids	4 Week Substance Use Scale (4WSUS)	/	Ayahuasca ceremonies focused on addiction	/		No effect		
			Cocaine	4 Week Substance Use Scale (4WSUS)	/	Ayahuasca ceremonies focused on addiction	/		Significant decreases		Yes
Johnson 2017	https://pubmed.ncbi.nlm.nih.gov/28095732/	Retrospective study	Smoking addiction	Fagerström Test for Cigarette Dependence (FTCD)	Fagerström Test for Cigarette Dependence (FTCD), Questionnaire on Smoking Urges (QSU)	Psilocybin, LSD, Ayahuasca	Moderate/high, mean of 2–5 lifetime uses	358	137 participants quitted, 100 reduced smoking and 121 stopped and then relapsed according to QSU scores	> − 1 year	Yes
Barbosa 2018	https://pubmed.ncbi.nlm.nih.gov/29740355/	Cross selectional study (ayahuasca users vs. national normative sample)	Alcohol use disorder	Brazilian version of the Substance Abuse and Mental Health Services Scale (SAMHSA)	Brazilian version of the Substance Abuse and Mental Health Services (SAMHSA), alcohol section of a questionnaire based on the WHO Research and Reporting Project on the Epidemiology of Drug Dependence	Ayahuasca	/	1947 (aya users in the study)	Current alcohol use disorder was significantly lower in the ayahuasca sample than the Brazilian norms. Regression analyses revealed a significant impact of attendance at ayahuasca ceremonies during the previous 12 months and years of aya consumption (rituals) on the reduction of alcohol use disorder.	/	/
			Tobacco use disorder	Brazilian version of the Substance Abuse and Mental Health Services Scale (SAMHSA)	Brazilian version of the Substance Abuse and Mental Health Services (SAMHSA), alcohol section of a questionnaire based on the WHO Research and Reporting Project on the Epidemiology of Drug Dependence	Ayahuasca	/		Current tobacco use disorder was significantly lower in the ayahuasca sample than the Brazilian norms. Regression analyses revealed a significant impact of attendance at ayahuasca ceremonies during the previous 12 months and years of aya consumption on the reduction of tobacco use disorder.	/	/
Barsuglia 2018 (no access to paper)	https://www.sciencedirect.com/science/article/abs/pii/S0079612318300931	Case report (SPECT neuroimaging included)	Alcohol use disorder	DSM IV	Non specified (cannot access paper)	5meo DMT/ibogaine	17.9 mg/kg ibogaine on day 1, 7 mg 5meo DMT on day 3	1	Cessation of alcohol use and reduced cravings at day 5 post-treatment, sustained at 1 month. Partial return on mild use at 2 months.	/	Yes but qualitative
Brown 2018	https://www.frontiersin.org/articles/10.3389/fphar.2018.00529/full	Observational study	Opioid use disorder	DSM-IV Opioid Dependence with Physiological Dependence	Addiction Severity Index (ASI), Lite version, 16-item Subjective Opioid Withdrawal Scale (SOWS)	Ibogaine	1,540 ± 920 mg	30	Significantly decreased ASIC scores were evident at all posttreatment time points for Drug Use (p < 0.001 at 1, 3, 6, 9, and 12 months). SOWS score had a mean reduction of 17.0 ± 12.5 points (t = 7.07, df = 26, p < 0.001) from pretreatment to after treatment.	/	Yes but qualitative
Davis 2018	https://pubmed.ncbi.nlm.nih.gov/29708042/	Retrospective survey	Alcohol use disorder	Survey question on being diagnosed with alcohol use disorder (no validated questionnaire)	A following question asked if their symptoms improved, got worse or stayed the same after 5meo dmt consumption	5meo DMT		22% of 505	66% reported a better change in AUD symptoms	/	Yes
			Drug use disorder (other than alcohol)	Survey question on being diagnosed with drug use disorder (no validated questionnaire)	A following question asked if their symptoms improved, got worse or stayed the same after 5meo dmt consumption	5meo DMT		33% of 505	60% reported a better change in SUD symptoms	/	Yes
Malcom 2018	https://www.semanticscholar.org/paper/Changes-in-Withdrawal-and-Craving-Scores-in-Opioid-Malcolm-Polanco/6a87a802a8bdfcfd126660f71e8c1e6b0353eab1	Observational study	Opioid use disorder	Diagnosis of OUD made by clinic physicians using DSM-5 criteria	Clinical Opioid Withdrawal Scale (COWS), subjective Opioid Withdrawal Scale (SOWS), Brief Substance Craving Scale (BSCS), Addiction Severity Index (ASI)	Ibogaine	18–20 mg/kg	50	COWS decreased pre- and post-ibogaine phases of the detoxification protocol, *F* (3, 47) = 18.71, (*p* < 0.01, *η*^2^ = 0.537), SOWS also decreased pre to post treatment,*F* (3, 45) = 11.24, (*p* < 0.01, *η^2^* = 0.428). Same for BSC, *F* (3, 45) = 32.80, *p* < 0.01, *η*^2^ = 0.69).	/	/
Mash 2018	https://www.frontiersin.org/articles/10.3389/fphar.2018.00529/full	Retrospective observational study (open label case series)	Opioid use disorder	DSM IV	Objective opioid withdrawal scale (OOWS), Heroin (HCQ-29) Craving Questionnaire	Ibogaine	8–12 mg/kg	102	Significant reductions in HCQ-29 scores at discharge and at 1 month follow up (*p* < 0.001), OOWS scores decreased individually but no stats reported	/	Yes but qualitative
			Cocaine use disorder	DSM IV	Cocaine craving questionnaire (CCQ-29)	Ibogaine	8–12 mg/kg	89	Significant reductions in CCQ-29 scores at discharge and at 1 month follow up (p < 0.001)	/	Yes but qualitative
Agin-Liebes 2019	https://pubmed.ncbi.nlm.nih.gov/33860184/	Retrospective survey	Alcohol use disorder	Mental health measure with question asking about alcohol misuse	The measure had a question asking if the alcohol use got better/stayed the same/worsened after mescaline consumption	Mescaline	/	72	Alcohol misuse got better in 48 participants	/	Yes
			Drug use disorder	Mental health measure with question asking about drug misuse	The measure had a question asking if the alcohol use got better/stayed the same/worsened after mescaline consumption	Mescaline	/	85	Drug misuse got better in 58 participants	/	Yes
Garcia-Romeu 2019	https://pubmed.ncbi.nlm.nih.gov/31084460/	Retrospective survey	Alcohol use disorder	DSM 5 (self reported), minority with no SUD but problematic use	Alcohol Use Disorder Identification Test-Consumption (AUDIT-C), the DSM-5 Substance Use Disorder Symptom Checklist (self reported), Alcohol Urge Questionnaire (AUQ)	LSD, psilocybin, ayauhasca	Moderate/high	343	Statistically significant reductions in self-reported drinks per week, AUDIT-C scores, and AUQ craving	4 months-10 years	Yes
Garcia-Romeu 2020	https://www.frontiersin.org/articles/10.3389/fpsyt.2019.00955/full	Retrospective survey	Cannabis use disorder	DSM 5 (self reported), minority with no SUD but problematic use	Drug Use Disorders Identification Test-Consumption (DUDIT-C), the DSM-5 Substance Use Disorder Symptom Checklist (self reported)	LSD (50%), psilocybin(30%), other (20%)	Moderate/high	166	Statistically significant reductions in self-reported DUDIT-C scores, and DUQ craving.	4 months-10 years	Yes
			Stimulant use disorder	DSM 5 (self reported), minority with no SUD but problematic use	Drug Use Disorders Identification Test-Consumption (DUDIT-C), the DSM-5 Substance Use Disorder Symptom Checklist (self reported), Drug Urge Questionnaire (DUQ)	LSD (40%), psilocybin(32%), other (29%)	Moderate/high	123	Statistically significant reductions in self-reported DUDIT-C scores, and DUQ craving.	4 months-10 years	Yes
			Opioid use disorder	DSM 5 (self reported), minority with no SUD but problematic use	Drug Use Disorders Identification Test-Consumption (DUDIT-C), the DSM-5 Substance Use Disorder Symptom Checklist (self reported), Drug Urge Questionnaire (DUQ)	LSD (40%), psilocybin(28%), other (33%)	Moderate/high	155	Statistically significant reductions in self-reported DUDIT-C scores, and DUQ craving.	4 months-10 years	Yes
Argento 2021	https://pubmed.ncbi.nlm.nih.gov/34758431/	Prospective cohort survey study	Opioid use disorder	Self reported daily use of of any illicit opioids (including heroin, fentanyl and nonmedical use of pharmaceu- tical opioids) in the last 6 months	/	Classic psychedelics	/	3813 participants at baseline, 1093 (29%) reported daily use of illicit opioids and 229 (6%) reported psychedelic use in the past 6 months	Over study follow-up after adjusting for a range of potential confounders, psychedelic use remained independently associated with a significantly reduced odds of subsequent daily opioid use (Adjusted Odds Ratio: 0.45; 95% Confidence Interval: 0.29–0.70)	1 day/ 6 months	/

### Classic psychedelics

Several retrospective and association studies have reported on the use of classic psychedelics including LSD, psilocybin, DMT, ayahuasca, and mescaline in individuals suffering from various addictions. A study by Pisano et al. (33) looked at 44,000 individuals with a history of illicit opioid use and found psychedelic use to be associated with a 27% reduced risk of past-year opioid dependence. A retrospective study of 358 individuals with smoking addiction who had 2–5 lifetime uses of moderate to high doses of either psilocybin, LSD, or ayahuasca found 137 participants quit, 100 reduced smoking, and the remaining 121 stopped but then relapsed (34), suggesting the use of classic psychedelics helped to reduce or quit smoking. Similarly, a series of retrospective surveys by Garcia-Romeu et al. (35, 36) found that moderate to high doses of classic psychedelics in over 700 individuals with DSM-5 self-reported alcohol, cannabis, stimulant, and opioid use disorder (OUD) was associated with statistically significant reductions in self-reported Drug Use Disorder Identification Test (DUDIT-C) scores and Drug Use Questionnaire (DUQ) craving.

Another retrospective study using data from the National Survey on Drug Use and Health (2015–1019), investigated the relationship between lifetime use of four classic psychedelic compounds and odds of past-year cocaine use disorder (CUD), in a representative sample of the U.S population (*n* = 214,505). Multivariate logistic regression analysis revealed that lifetime peyote use reduced the odds of CUD by 50% (OR: 0.47) as well as the odds of CUD criteria [OR range: 0.26–0.47; (37)].

A prospective cohort survey was conducted by Argento et al. (38) study in 1093 individuals with daily use of illicit opioids in the last 6 months. In the study follow-ups, after adjusting for a range of potential confounders, psychedelic use in these individuals remained independently associated with significantly reduced odds of subsequent daily opioid use (OR, 0.45; 95% CI: 0.29 to 0.70).

For a full review of studies exploring the relationship between classic psychedelics and alcohol use in humans see Calleja-Conde et al. (39), which shows a positive association in reductions in alcohol addiction as seen observational studies.

### 5-MeO-DMT

5-MeO-DMT is a short-acting naturally occurring tryptamine that is produced by several plants and the Sonoran desert toad [*Incilius alvarius*; (40)]. There has been one retrospective survey to date on the use of 5-MeO-DMT in treating individuals with alcohol and other drug use disorders. Of the 1010 participants surveyed with alcohol and drug addiction, 66% of the alcohol and 60% of the drug addiction group reported an improvement in their condition (41).

### Ayahuasca

One cross-sectional study comparing ayahuasca users versus a normative sample found both significantly lower last year and last month alcohol consumption (42). Another study in polydrug users reported significantly lower scores in addiction severity index subscales and current drug use (except for cannabis) in ayahuasca consumers [*p* < 0.05; (43)]. The latter study noted the possible therapeutic role of the church and the community in contributing to these effects. In a cross-sectional study conducted in Brazil, current alcohol and tobacco use disorder (TUD) was found to be significantly lower in the ayahuasca sample than in Brazilian normative samples. Regression analyses revealed a significant impact of attendance at ayahuasca ceremonies during the previous 12 months and years of ayahuasca consumption on the reduction of incidence of both alcohol and TUD (44).

### Mescaline/peyote

Mescaline is a naturally occurring alkaloid found in cacti, mainly in the peyote cactus (*Lophophora williamsii*) and in the cacti of the *Echinopsis* genus (45). The Native American Church have a history of using peyote in the context of ritualized sacramental practices to aid recovery from addiction and substance misuse. Several anthropological studies have documented its use and beneficial anti-addictive effects in these settings in the US (46–48). One retrospective survey in individuals with alcohol and ‘drug use disorders’ found 48 out of 72 with alcohol addiction and 58 of 85 with ‘drug use disorder’ improved following ingestion of mescaline (49).

### Non-classic psychedelics

#### Ibogaine

As noted from historical studies previously, ibogaine showed early promise in neuropsychiatry, though due to concerns related to cardiotoxicity and cerebellar ataxia, clinical research in this field ceased. However, an abundance of real-world evidence has accumulated supporting its potential, particularly for opiate addiction where it is an approved medicine for this indication in New Zealand. One observational study in 32 individuals with opiate dependence reported significant reductions on several opioid withdrawal scales at 12-, 24- and 36-h post ingestion of 10 mg/kg ibogaine [*p* < 0.05; (50)]. Similarly, a study of 14 opiate dependent individuals given an average of 31 ± 8 mg/kg detected significant decreases in subjective opiate withdrawal scores from 25 ± 13 pre-ibogaine to 14 ± 14 at 12–24 h post-ibogaine (*p* = 0.015). Further, this study reported significant decreases in scores on the addiction severity index from baseline to 12-months follow-up [*p* < 0.002; (51)]. A host of other observational studies corroborated these findings (52, 53). Moreover, in an open-label case series of 89 individuals with CUD, scores on the cocaine craving questionnaire (CCQ-29) were significantly reduced at discharge, which was sustained at 1 month follow-up after administration of 8–12 mg/kg of ibogaine (54).

Intriguingly, one Single Photon Emission Computed Tomography (SPECT) neuroimaging case study in an individual with alcohol use disorder (AUD) combined ibogaine at day one with 5-MeO-DMT at day three and found cessation of alcohol use, reduced cravings, and improved moods at day five post-treatment. The effect was sustained at 1 month and then a partial return to mild use followed at 2 months. Increases in brain perfusion were seen in the left putamen, and right insula, as well as temporal, occipital, and cerebellar regions, compared to the patient’s baseline scan. These regions have been implicated in the pathology of alcohol addiction, as assessed with functional and molecular neuroimaging, and are key hubs of the mesocorticolimbic dopaminergic reward system (55).

In sum, naturalistic and observational research spanning survey-based studies, retrospective data analysis and prospective case series generally indicate a positive association between the use of psychedelic substances and reductions in the incidence of addiction and substance abuse or misuse.

### Modern-era clinical trials

Currently several clinical trials are underway exploring the therapeutic use of psilocybin, MDMA, ketamine, and ibogaine in the treatment of alcohol, tobacco, opiate, methamphetamine, cocaine, and gambling addiction. In this section, we will describe some of these emerging findings and explore the future direction of this research. A summary of these studies can be found in Table 2.

**Table 2:** Modern day clinical interventional studies of classic and non-classic psychedelics in addiction.

Author	Diagnosis	Trial and treatment	*N*	Outcome
(56)	Alcohol dependence (DSM-IV)	Open-label single arm proof of concept 2× high doses of Psilocybin (0.3 mg/kg and 0.4 mg/kg) + 7× sessions of motivational enhancement therapy 3× preparation sessions, 2× integration sessions	10	Psilocybin rapidly and significantly reduced the percentage of drinking days and the number of heavy drinking days in this proof-of-concept study, with effects lasting up to 36 weeks post intervention. In secondary analyses psilocybin significantly reduced craving scores (50% reduction at 36 weeks follow up). Correlations between the acute effects of psilocybin predicted changes in drinking, craving and self-efficacy.
(57)	Nicotine addiction (minimum 10 cigarettes a day multiple unsuccessful past quit attempts, and still desire to quit smoking)	Open-label single arm proof of concept 2-3× administrations of psilocybin (20 mg/70 kg – moderate & 30 mg/70 kg – high). 15-week smoking cessation treatment protocol	15	Biomarkers assessing smoking status, and self-report measures of smoking behavior, demonstrated that 12 of 15 participants (80%) showed seven-day point prevalence abstinence at 6-month follow-up. 11/12 of these individuals reported cessation after just the first psilocybin administration.
(68)	Alcohol use disorder (DSM-5 moderate to severe AUD or DSM-IV alcohol dependence)	Double-blind placebo-controlled Phase IIa 3× 0.8 mg/kg intravenous ketamine infusion or saline infusion plus Psychological or Alcohol education	96	Significantly greater number of days abstinent from alcohol in the ketamine group compared with placebo group at 6-month follow-up (mean difference = 10.1, 95% CI = 1.1, 19.0), with the greatest reduction in the ketamine plus therapy group compared with the saline plus education group (15.9, 95% CI = 3.8, 28.1). There was no significant difference in relapse rate between the ketamine and placebo groups.
(58)	Alcohol use disorder (DSM-5)	Open-label single arm proof of concept 2× 187.5 mg MDMA-assisted psychotherapy sessions community based alcohol detox, 8x week recovery-based therapy	14	MDMA treatment was well tolerated by all participants. No unexpected adverse events were observed and psychosocial functioning improved across the cohort. At 9 months post detox, the average units of alcohol consumption by participants were 18.7 units per week compared to 130.6 units per week before the detox. This results compare favorably to outcomes from patients undergoing similar community based alcohol detox
(59)	Alcohol use disorder (DSM-5)	Double-blind randomized clinical trial, participants were offered 12 weeks of manualised psychotherapy and randomly assigned to receive psilocybin (25–40 mg/70 kg) vs. diphenhydramine (50–100 mg) during 2 day-long medication sessions at weeks 4 and 8. Outcomes assessed over a 32-week double-blind period following. Study medications were psilocybin, 25 mg/70 kg, vs. diphenhydramine, 50 mg (first session) Psychotherapy included motivational enhancement therapy and cognitive behavioral therapy.	93	Percentage of heavy drinking days during the 32-week double-blind period was 9.7% for the psilocybin group and 23.6% for the diphenhydramine group, a mean difference of 13.9%; (95% CI, 3.0–24.7; *F*_1,86_ = 6.43; *p* = 0.01). Mean daily alcohol consumption (number of standard drinks per day) was also lower in the psilocybin group. No serious adverse events among participants who received psilocybin.

### Psilocybin

Psilocybin is the main psychoactive compound in ‘magic mushrooms.’ After the observation in 1953 in Mexico of ritual practices involving the ingestion of such mushrooms, psilocybin was chemically characterized and synthesized in 1958. Since then psilocybin has been explored for therapeutic uses across a range of psychiatric disorders (60).

The strong findings of earlier work with psychedelic therapy for alcoholism have led to a modern replication study of psilocybin-assisted treatment for alcohol dependence. A pilot study by Bogenschutz et al. (56) included 10 volunteers with a DSM-IV diagnosis of alcohol dependence. Participants were given seven sessions of Motivational Enhancement Therapy [MET; a structured approach using principles derived from motivational interviewing; (61)] and two high doses of psilocybin were administered at 0.3 and 0.4 mg/kg sequentially. Psilocybin was found to rapidly and significantly reduce the percentage of drinking days and the number of heavy drinking days in this proof-of-concept study, with effects lasting up to 36 weeks post-intervention. Psilocybin significantly reduced craving scores by 50% and this was significant at 36 weeks follow-up. There were also associations between the acute effects of psilocybin as measured by the mystical experiences questionnaire (MEQ), predicting changes in drinking, craving, and self-efficacy. These findings were recently replicated in a phase II double-blind randomized clinical trial with 93 participants (59) which found the percentage of heavy drinking days was 10% in the psilocybin group during the 32-week double-blind period and 24% for the control diphenhydramine group, a mean difference of 14%; [95% CI, 3.0–24.7; *F*(1,86) = 6.43; *p* = 0.01]. Mean daily alcohol consumption (number of standard drinks per day) was also significantly lower in the psilocybin group. This study is now being replicated in larger phase III studies across multiple centres as part of the pathway to marketing authorisation for this indication.

Psilocybin has also shown early promise in TUD in a study led by Johnson et al. (57) at Johns Hopkins University. This single-arm open-label study followed 15 treatment-seeking participants through a 15-week intervention involving CBT for smoking cessation and two to three administrations of psilocybin. These participants had a mean of six previous quit attempts, smoking an average of 19 cigarettes a day for 31 years preceding the study. Results were quantified using both subjective questionnaires and confirmed biochemical assays, exhaled carbon monoxide and urinary cotinine levels. Twelve out of the 15 (80%) participants showed biochemically confirmed abstinence at the 6-month follow up and 11/12 of these individuals reported cessation after just the first psilocybin administration. This preliminary study showed a substantially higher rate of cessation than the best available behavioral or pharmacological interventions; typically less than <35% abstinent at 6 months post-treatment (62). Similar to the study on alcohol dependence, higher mystical experience scores were correlated with better outcomes (63). A larger phase II study (NCT01933994) is now underway which has received the first governmental funding of a modern-era psychedelic trial in the US since the ban in 1971.

A double-blind RCT is also being conducted in CUD at the University of Alabama with early, non-published results claiming the first 10 patients experienced higher life satisfaction, less depression, and more abstinent days from cocaine than those in the placebo arm (64). There is also an interventional, randomized trial in 30 adults with methamphetamine addiction who will be given either two doses of 25 and 30 mg psilocybin, respectively, or treatment as usual, with results from this study due in June 2024 (65). In a small, open-label, safety study, psilocybin is also being investigated for its interaction in individuals with OUD who are being maintained on a buprenorphine-naloxone formulation (66). Such work is important to understand possible drug–drug interactions and to ensure the safety of participants with OUD who are often maintained on these medications. Further, at our own Imperial College London centre, we are initiating an open-label study into the neuromechanistic and clinical effects of psilocybin in the treatment of detoxified OUD individuals, the first of its kind in this specific group.

### Ketamine

The quasi-experimental case-study data from Russia (27) has prompted the modern clinical exploration ketamine in RCTs. A recent US study in 40 outpatients with alcohol dependence randomized to either a single infusion of ketamine (0.71 mg/kg) vs. midazolam as an active placebo, alongside motivational enhancement therapy, demonstrated that ketamine significantly increased the likelihood of abstinence, delayed the time to relapse, and reduced the likelihood of heavy drinking days compared with midazolam (67). Another recent double-blind placebo-controlled phase II clinical trial in the UK included 96 patients with AUD randomizing patients to four possible treatment arms; ketamine or placebo infusion and mindfulness psychotherapy or psychoeducation, respectively. The treatment was well tolerated and the most positive effects were demonstrated in the group receiving three infusions of 0.8 mg/kg ketamine plus psychotherapy, who had more days of abstinence at 6 months follow-up than the placebo infusion plus psychoeducation group (68). The added value of this study is that it suggests the possible adjunctive therapeutic effect of psychotherapy combined with ketamine.

There has been limited neuroscientific research into the mechanism of ketamine in addiction, though one study (69) found that ketamine administration immediately following maladaptive reward memory retrieval (beer cue-exposure) resulted in significant decreases in drinking volume, drinking enjoyment and urge to drink versus those receiving ketamine or memory retrieval alone. This demonstrates pharmacological interference with memory reconsolidation may allow overwriting of maladaptive drinking memories with clinical benefits.

### MDMA

MDMA is a psychoactive drug developed by Merck at the beginning of the 20th century, exerting its primary neurobiological effect through the blockade of the monoaminergic reuptake transporters (70). It has been investigated clinically for its ability to enhance psychotherapy [for review see (71)], specifically in couples counseling (72), as a treatment for PTSD (73, 74), and for AUD (58). In AUD, a proof-of-concept study was conducted to assess its safety and tolerability in 14 patients. MDMA was found to be well tolerated by all participants and there were no reports of severe adverse events. Furthermore, the average alcohol consumption of participants at 9 months post-detoxification was 18.7 units per week versus 130.6 units before pre-trial detoxification (58). Further testing of MDMA for AUD is currently underway led by the biotechnology company AWAKN Life Sciences and another trial looking at MDMA to treat co-occurring PTSD and OUD after childbirth has been registered (NCT05219175).

### Ibogaine

There are several clinical trials currently registered to investigate Ibogaine in the treatment of methadone detoxification in opioid dependence (NCT04003948), and in opioid withdrawal (NCT05029401), with ambitions to assess safety, tolerability and treat-to-target dose. A further double-blind placebo-controlled escalating dose phase II study is also registered in the treatment of AUD. These studies are ongoing with no currently published results (NCT03380728). Currently, a clinical-stage pharmaceutical company called DemeRx are developing specific metabolites of ibogaine and these are currently undergoing clinical development. Another molecule in development is Tabernanthalog (TBG) which is a functional analog of Ibogaine recently developed at the University of California (75). So far, preclinical research has found that TBG increased dendritic arbor complexity in a 5-HT_2A_R dependent manner in embryonic cortical neurons, selectively reduced alcohol consumption, and acutely reduced heroin-seeking behavior and cue-induced relapse in rodent models of human binge drinking and heroin self-administration (76). The effect of cumulative doses of TBG on heroin relapse in a preclinical heroin model has been internally replicated (75), however, the compound still needs to go through phase I safety and tolerability studies and phase II dose-finding and proof of mechanism studies before being tested in larger phase III clinical trials.

Modern-era human clinical research of psychedelic therapy in addiction has allowed for the assessment of several compounds on clinical outcomes and psychological mediators of treatment response. These studies have an advantage over historical studies which were not conducted to the same rigorous scientific standards that current clinical trials are held to. These early results have demonstrated efficacy and safety and in the next few years, larger phase III clinical trials will begin with the purpose of gaining marketing authorisation.

### The future: how biomedical and translational neuropsychopharmacology can optimize the development of psychedelic therapies for addiction

In the following sections, we will discuss how advancements in biomedical science, with a particular focus on in-human neuropsychopharmacology studies, have been instrumental in our understanding of the mechanisms and processes of addiction. We will discuss how these tools can be leveraged to optimize the development of psychedelic therapies profitably for addiction and how they lend themselves to personalized medicine and precision psychiatry efforts. It is important to note that the literature on human neurobiological psychedelic mechanisms in addiction is sparse.

### Translational neuropsychopharmacology to optimize psychedelic therapy for addiction

Translational neuropsychopharmacology describes utilizing human neuroimaging techniques specifically in the development of novel psychopharmacological interventions for psychiatric populations. Neuroimaging provides researchers with unparalleled insight into the pathobiology of disorders such as addiction, allowing for the identification of putative functional and molecular biomarkers. These tools are also used to assess the effects of novel interventions, such as psychedelics, on addiction biomarkers and their relationship with clinical and behavioral outcomes.

### Conceptual framework of addiction and brain function

**Figure 1:**
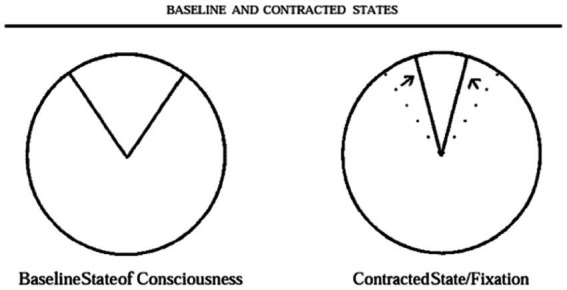
Baseline and contracted states.

Ralph Metzner, one of the key figures in the first wave of psychedelic research, in a seminal theorem titled ‘Addiction and transcendence as altered states of consciousness,’ describes addiction as a narrowed state of consciousness, depicted as a 15-degree window of a 360-degree circle (77). He writes ‘many addicts crave a certain experience, a state of consciousness’ and that addiction should be viewed as a ‘complete contraction and fixation of consciousness.’ In this state, an individual’s attention is singularly fixed on ‘the object of desire, the craved sensation, the bottle, or the pipe, to the exclusion of other aspects of reality, other segments of the total circle.’ A schematic from this paper is presented in Figure 1.

**Figure 2:**
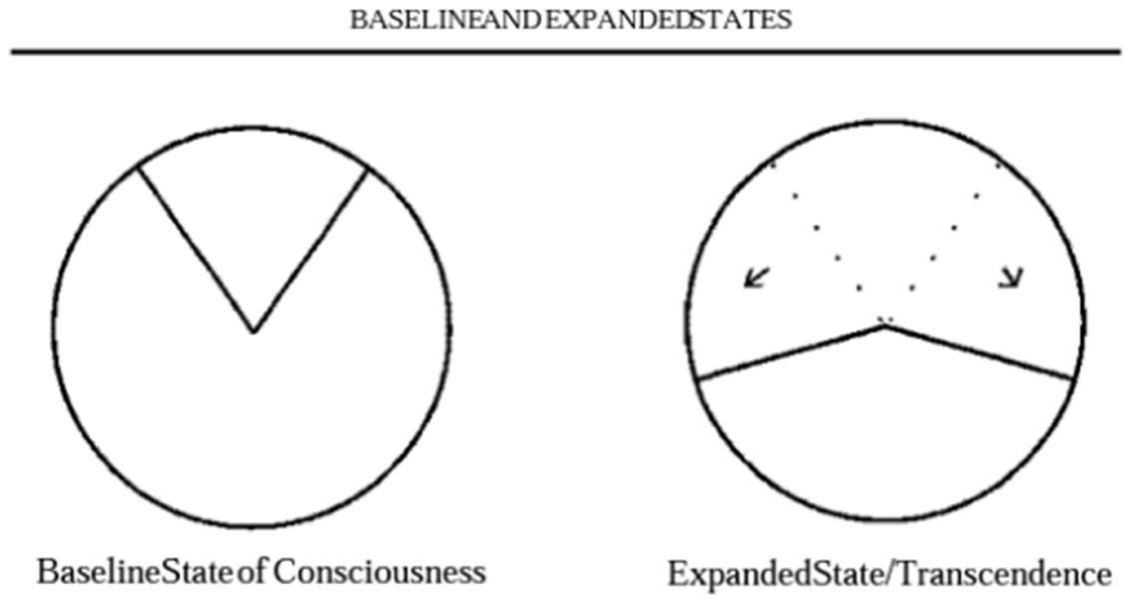
Baseline and expanded states.

He then describes that in states of transcendence, where there is heightened awareness, such as seen under the influence of LSD or psychedelics, there could be a ‘master resetting of perspective on the field of conscious awareness.’ He argues that for individuals to thrive, they need to sometimes employ expanded awareness, and a key failure in addiction is the inability to take onboard new information or acquire novel salient information for survival, possibly due to the hijacking of the brain’s reward circuits. This can be represented as an increase from the baseline state of consciousness to a wider arc of >180 degrees of conscious awareness as depicted in Figure 2.

This theoretical framework preceded many of the advancements in identifying human neuroimaging biomarkers in addiction. However, Metzner’s theory portrays findings from human addiction neuroimaging studies that have demonstrated brain impairments in cognitive, emotional and memory systems and reduced salience network function (78) which are thought to be mediated by neurotransmitter deficits (79) in individuals with addiction. Thus, the described diminished arc of consciousness can be considered to reflect the neurobiological features seen in patients with this disorder. Whilst these tools can assess pathobiology they can also investigate the effects and impacts of interventions such as psychedelic therapy. Could it be that psychedelic therapy may break down contracted conscious awareness in addiction and re-broaden the brain’s salience networks and restore molecular deficits?

The availability of advanced functional and molecular neuroimaging tools allows us to directly test the hypotheses that psychedelics may restore:

i. Impairments in brain function related to reward processing, salience attribution, impulsivity and inhibitory control and emotional regulation through the re-broadening of hijacked reward networks
ii. Neurotransmitter deficits and dysfunction that have been identified as biomarkers of addiction including the dopamine, opioid and serotonin systems
iii. Deficits in cortical neuroplasticity

These findings would help us to identify for the first time the brain mechanisms mediating the relationship between psychedelic therapy and clinical, behavioral and neurocognitive outcomes and how they relate to relapse vulnerability in individuals with addiction. In the following sections, we aim to introduce the known biomarkers of addiction and propose a series of experimental techniques to explore the role of psychedelic therapy on these.

### Functional magnetic resonance imaging (fMRI)

FMRI techniques have been developed to probe the neural responses to cognitive and psychological tasks, external stimuli, and pharmacological or behavioral challenges. FMRI measures brain activity via the blood oxygenation level-dependent (BOLD) signal, providing an indirect measure of brain activity that can be examined in response to neurocognitive tasks or at rest. In the context of addiction, several well-validated fMRI paradigms have been developed that assess the neural responses to reward, punishment, salience attribution, emotions, memory, cognitive and executive function and their association with clinical outcomes and relapse vulnerability. In particular, our group have previously successfully developed an fMRI platform that assessed novel candidate drugs on the aforementioned key relapse pathways known to be dysfunctional in individuals with addiction using task-based fMRI (80). We suggest that the development of novel pharmacotherapies for addiction, such as psychedelic therapy, is best conducted through the investigation of these interventions using such fMRI platforms, to establish brain mechanisms related to addiction and relapse. Such an approach, we hope, will allow for more individualized and effective treatments to be developed and may have value in predicting treatment response (81, 82). In the following sections, we will consider how fMRI paradigms can be employed to investigate the effect of psychedelic therapy on addiction-related processes and treatment response in the context of clinical studies.

### Reward processing

There is significant evidence for dysfunction in reward processing in addiction. Increases in activity in the ventral striatum (VS) have been found in response to observed or actual drug reinforcement, and to monetary rewards in both individuals with addiction and non-addicted populations (83), with modulation in the activity of the prefrontal cortex (PFC) and orbitofrontal cortex (OFC) which are involved in weighing up benefits and costs and motivation for rewards (84). One of the most common fMRI tasks employed in addiction populations which measures neural responses to monetary reward processing, is the monetary incentive delay task (MIDT) (85); which has identified pathophysiological reward processing in individuals with addictions. Meta-analyses from studies employing this paradigm have generally found hypo-functioning of the VS during the anticipation of reward in patients with several subclasses of addiction versus matched healthy controls (86). VS activation to this task has also been used to assess the functional effects of drugs being developed as potential addiction medicines, including dopamine D_3_ receptor (DRD_3_) antagonists (87) and already licensed drugs such as naltrexone (88) and nalmefene (89). These studies were designed to assess if these novel drugs (DRD_3_) modulated reward processing in individuals with addiction in the same way as approved drugs (nalmefene and naltrexone) and therefore were viable candidates for clinical development. There is also evidence that VS hyporesponsivity to the MIDT ‘normalises’ over time with abstinence and recovery (90), and hence this task could be used in a longitudinal study design to investigate the enduring effects of novel interventions, such as psychedelic therapy, on brain-function and its association with clinical outcome.

There has been limited in-human neurobiological research on the effects of psychedelics on reward processing, though one recent study in healthy individuals examined the effects of two single, low doses of LSD compared to placebo on measures of reward feedback processing. These results constitute the first evidence that low doses of LSD increase reward-related brain activity in humans (91) and suggest a possible therapeutic mechanism of action for patients with addiction who present with reward deficiencies (92).

### Cue-reactivity

FMRI studies have observed dysregulated neural responses to video and photo cues in individuals with addiction when compared with matched healthy control subjects. Particularly hyper- and hypo-activations of salience, attentional, executive, and memory networks have been observed in response to addiction-salient versus naturally rewarding or non-salient video and photo cues (78). Greater responses to addiction-related stimuli and craving are observed in patients with addiction and this represents aberrant incentive sensitisation which is theorized to lead to maladaptive drug-taking behaviors (93). Further, aberrant fMRI cue-reactivity has also been demonstrated in individuals with gambling disorders (GDs) when compared with matched controls, showing greater gamblers to have greater reactivity to gambling cues in the left insula and the anterior cingulate cortex (ACC) and with craving to gamble correlated positively with gambling cue-related activity in the bilateral insula and ventral striatum (94). As a behavioral addiction, GD provides an opportunity to characterize addictive processes without the potentially confounding effects of chronic excessive drug and alcohol exposure, and thus patterns of dysregulated brain activity may represent central pleitropic biomarkers of addiction and be a target for the development of novel interventions. Cue-reactivity fMRI has also shown utility in being able to predict addiction severity, risk of relapse and treatment outcome (95) and has been leveraged to develop novel therapeutics in addiction (96). To date, there have been no published literature relevant to cue-reactivity in addiction populations being treated with psychedelic therapy. Hence, fMRI cue-reactivity paradigms provide an ideal platform to assess the neurobiological effects of psychedelic therapy on reward and motivational systems in addiction. Integration of fMRI cue-reactivity paradigms into clinical studies in a pre- vs. post-psychedelic design is recommended to assess changes in brain function and its association with craving and longer-term clinical outcomes, such as relapse. Further, it would also be intriguing to understand how psychedelics acutely modulate salience network (SN) activation in patients with addiction, and how this relates to task-based cue-reactivity to understand the acute effects on psychedelics on reward processing in clinical populations.

### Emotional and social processing

Addictions have been consistently linked to strong negative affective states, withdrawal and emotional dysregulation with several regions of the brain, including the amygdala and frontal cortex, found to play a role in driving these responses (97). Several fMRI tasks have been developed to probe alterations in these domains in patients with addiction. These include the evocative images task [EIT; (98)] which is used to assess responses to aversive and stressful stimuli and to assess amygdala reactivity, which is known to be dysfunctional in individuals with addictions (99). Indeed the EIT has been used to assess the effects of novel interventions, such as a DRD_3_ antagonist, on brain mechanisms relevant to emotional regulation in abstinent drug-dependent individuals (100) demonstrating its sensitivity to pharmacological modulation. Interestingly, psilocybin has been found to decrease connectivity from the amygdala to the primary visual cortex during threat processing (101) and reduce connectivity between the amygdala and the striatum during angry face discrimination (102). These data demonstrate that psilocybin modulates the amygdala to adaptively process fear responses. This lends support to theory that psychedelics allow individuals to process threat and actively engage with their emotional environment as opposed to adopting avoidant coping mechanism strategies to reduce stress, such as drug seeking in patients with addiction.

Additionally, both LSD and psilocybin have been found to acutely increase emotional empathy and sociality (103, 104) in healthy individuals. There is neurobiological evidence that psilocybin mediates the effects of reduced social rejection processing and the feeling of social exclusion through decreasing activity in the dorsal ACC and the middle frontal gyrus, which are key regions for social pain processing and are implicated in the neurocircuitry of stress responsivity which are known to be dysregulated in addictions (97). These findings demonstrate that, under the acute effects of psilocybin, there is a reduction in social pain processing and changes in self-experience associated with disengaging the amygdala from maintaining threatening states.

Further, such paradigms have been used to assess the sub-acute brain changes from psychedelic therapy and its relationship to treatment outcomes in clinical populations. In patients with treatment-resistant depression (TRD), psilocybin increased amygdala responses to fearful faces 1 day after a psilocybin session and this correlated with clinical outcome (105). A follow-up analysis demonstrated that decreased ventromedial-PFC-right amygdala functional connectivity during fearful face processing post- (versus pre-) treatment was driving these effects and was associated with changes in rumination at one-week post-dose (106). Equally, such a strategy could feasibly be employed in clinical studies with addiction given the transdiagnostic psychological and neural domains that these tasks engage.

Another method to probe both changes in emotive responsivity and reward/anhedonia processing is through music listening fMRI tasks. In one recent study, participants with TRD were played music pre- vs. post psilocybin therapy and were assessed on changes in subjective outcomes and nucleus accumbens (NAc) activity. Results revealed increases in music-evoked emotion following treatment that correlated with reductions in anhedonia post-treatment with associated changes in NAc functional connectivity (107). These results highlight another task-based technique to probe dysfunctional neurocognitive and neurobiological domains in addiction.

Collectively these lines of evidence support that psychedelics may facilitate a restoration to adaptive emotional and social processing strategies which appear to be mediated by brain effects involving the amygdala and associated stress neurocircuitry. So far psychedelic studies have addressed both the acute effects of psilocybin on emotional and social regulation and stress in healthy populations and the sub-acute changes in emotional processing and the brain effects that relate to clinical response in depression. To date, these paradigms have not yet been tested in patients with addiction undertaking psychedelic therapy and are warranted given the relevant overlap in the addiction-specific neurobiological and psychological features that these tasks explore.

### Cognitive flexibility, impulsivity and inhibitory control

Behaviors elicited by individuals with addiction can be characterized as impulsive, which describes the lack of inhibitory control and manifests as non-premeditated action (108). Increases in trait impulsivity have been consistently found in individuals with addictions compared to healthy controls when using validated clinical impulsivity scales (109). Choice impulsivity has also been found to be related to altered neural processing in regions of the reward system such as the VS, ACC, OFC and dorsolateral prefrontal cortex (DLPFC) (110). Motor inhibition, a way to measure prepotent inhibitory control, can also be assessed using tasks such as the Go/no-go task and has been used to assess the brain effects of naltrexone in abstinent alcohol and poly-substance dependent individuals, demonstrating its amenability to pharmacological interventions (111). Hypo- and hyper-activation in response to these tasks have been able to differentiate those currently using substances and predict the likelihood of relapse (112). With further development, this could be a plausible future stratification and prognostic marker to guide clinical decision-making for engagement with treatments such as psychedelic therapy.

The PFC has long been implicated in addiction with models such as Impaired Response Inhibition and Salience Attribution (IRISA) which summarizes the dysfunction of this brain region (113) and identifies it as a target to test novel treatments. As such, these imaging paradigms can feasibly be adopted into studies of the effects of psychedelics on addiction processes related to dysfunctional executive and cognitive control mediated by PFC-striatal connectivity.

There is some evidence to suggest psychedelics can modulate PFC and cognitive function in humans. Some recent work in patients with depression has shown psychedelic therapy to impact cognitive flexibility, as measured by perseverative errors on a set-shifting task, and its association with neural dynamic functional connectivity (dFC) and neurometabolite concentrations. The findings demonstrate how baseline dFC of the ACC was predictive of changes in cognitive flexibility post-treatment (114) and suggest that psychedelic therapy could equally target these transdiagnostic deficits in cognitive and neural flexibility that are observed in addicted populations.

We suggest fMRI paradigms such as the Set-shifting task (114), the Kirby delay discounting task (115), the Go/no-go task (116), the Iowa gambling task (117) and the Stop-signal task (118) should be used in a within-subject design, to test the change in functional and neural processing pre- vs. post-psychedelic therapy in cognitive and executive function. We recommend that the post-therapy fMRI scan takes place at least 7 days post-psychedelic therapy to allow for purported frontal neuroplastic changes to occur which may underlie the observed effects (119). We do not conduct fMRI tasks related to these domains under the acute influence of psychedelics as they are known to transiently impair elements of cognitive function (120) and would not provide valid clinically translational results. This design would enable us to assess the relevant sub-acute and longer-term neuromodulation of these domains and its association with treatment response and risk of relapse.

### Task-based fMRI study designs in psychedelic therapy for addiction studies

We have proposed the use of FMRI tools above that will help to explore functional biomarkers relevant to addiction processing covering reward, emotional and social processing and cognitive function. We believe these tasks are best conducted on a platform where multiple domains can be investigated in a single scanning session in patients with addiction either;

a) Under the acute influence of psychedelics to see the immediate neuropsychopharmacological effects on domains limited to; reward processing, cue-reactivity

Key contrasts: win-anticipation > neutral anticipation (MIDT); addiction salient cue > neutral cue

b) In a pre- vs. post-psychedelic therapy within-subject design to assess the sub-acute and longer-term changes in brain function in response to fMRI tasks exploring cognitive flexibility, impulsivity and inhibitory control and its relation to psychological and behavioral scales and clinical outcomes associated with addiction such as cognitive function and relapse. We further recommend reward, social and music processing and cue-reactivity to be incorporated into this type of study design to see the longer-term impacts psychedelics have in these central domains linked to addiction.

Key contrasts: (Cognitive errors) pre- psychedelic therapy > (Cognitive errors) post- psychedelic therapy

(Aversive stimuli) pre- psychedelic therapy > (Aversive stimuli) post- psychedelic therapy

(Emotive stimuli/music valence/arousal) pre- psychedelic therapy < (Emotive stimuli/music valence/arousal) post- psychedelic therapy

(Social processing) pre- psychedelic therapy < (Social processing) post- psychedelic therapy

Key brain regions: DLPFC, ACC, posterior cingulate cortex (PCC), OFC, PFC, VS, NAc, ventro-medial PFC, amygdala, SN, DMN, executive networks (EN).

### Resting state functional connectivity (RSFC)

RSFC measures the temporal correlation of spontaneous BOLD signals among spatially distributed brain regions, with the assumption that regions with correlated activity form functional networks. This approach has commonly been used in psychiatric and addiction investigations to assess the neurobiological basis of these disorders (121). Here, we will explore the currently available evidence for how psychedelics may induce a re-broadening of salience through the lens of network neuroscience. We will address the fundamental roles of intrinsic large-scale brain networks in addiction, crucially the DMN and the SN, and their potential as targets for accentuating psychedelic-induced perspective change and therapeutic efficacy in addiction. Where there are gaps in the current literature, we will offer examples of testable RSFC neuroimaging analyses that are able to probe the putative mechanisms of action of psychedelics in modulating addiction processes related to brain network dysfunction.

RSFC has played a fundamental role in revealing the mechanisms underpinning cognitive dysfunction and abnormal salience attribution to drugs and drug-related stimuli that is typical in addiction. This malfunction may arise from dysregulation in predominantly ‘mesocorticolimbic’ reward circuitry, both in resting-state and during the presentation of salient cues (122). The majority of prior work has focussed on specific regions of the dopaminergic midbrain and limbic system (e.g., striatum, ventral tegmental area, hippocampus, amygdala) and identified these areas as key contributors to addictive behavior (97). However, with developments in our understanding and methodologies in circuit neuroscience, our understanding of the brain from a systems perspective has led to an awareness of the impact of maladaptive connectivity in large-scale functional resting-state networks in addictive disorders (123).

Aberrant patterns of functional connectivity in the DMN have been observed across a number of addiction disorders, which have been associated with craving and relapse, stemming from impaired self-awareness, negative emotions and ruminations (124). In addicted individuals, RSFC of the anterior DMN (e.g., mPFC), which participates in the attribution of personal value and emotional regulation, tends to be decreased (125), whereas RSFC of the posterior DMN (e.g., PCC), which directs attention to the internal world, tends to be increased (126), indicating the influence of misplaced attentional resources in addiction. Further, in individuals with addiction, the connectivity within the DMN may be altered, leading to decreased coordination between different regions within the network (127). This decreased coordination may be associated with alterations in executive functioning and negative self-referential thinking, which can increase the risk of relapse (127).

Several recent reviews note how across psychedelics there is a consistent acute disruption in resting-state connectivity within the DMN and increased functional connectivity between canonical resting-state networks, see (128, 129). The role of DMN modulation has been proposed as a central neurobiological locus mediating the cognitive and therapeutic mechanism of psychedelics, although it is still unclear how central the DMN is to the therapeutic potential of classical psychedelic agents in patients with addiction.

In addition to the DMN, the SN has also been heavily implicated in the psychopathophysiology of addiction. The SN is comprised of structurally and functionally connected brain regions with cortical areas including the anterior insula and ACC, as well as subcortical and limbic structures such as the amygdala, VS and thalamus (130, 131). This network of regions is considered to support one’s socioemotional functions by integrating visceral and sensory information (132, 133), as well as playing an intrinsic role in directing one’s attention toward salient stimuli (134). Notably, there is a significant anatomical overlap between the SN and the mesocorticolimbic system, with recent preclinical work indicating that the firing of mesolimbic dopamine neurons may activate nodes of the salience network (135).

The SN is receiving increasing amounts of interest in psychedelic research, with evidence revealing that psychedelics acutely increase activity in the ACC, a key hub in the SN (136, 137). Conversely, decreased within-salience network coupling was observed during the acute effects of psilocybin (138), which was associated with “ego-dissolution” – or the feeling that the border between oneself and the external world is dissolving (139). Similarly, increased salience network entropy levels have been observed during the acute effects of LSD (140). This may indicate alterations in salience network activity which may facilitate the re-establishment of healthy patterns of brain functional inter-connectivity. A placebo-controlled study of psychedelic-naive healthy individuals given ayahuasca revealed increased ACC connectivity within the SN, decreased PCC connectivity within the DMN and increased connectivity between the SN and DMN 1 day after ayahuasca (131). Intriguingly, increased global coupling and functional connectivity between SN and DMN nodes have been found under acute psilocybin administration (141) but future work, ideally combining multimodal molecular and functional neuroimaging (142), is needed to elucidate how mid- and long-term functional interactions of both networks are changed by psychedelic therapy in populations with addiction and how these relate to prognosis.

Recent work from our group at Imperial College London found changes in brain modularity. Brain network modularity is a measure of the extent to which the brain’s inter-regional functional connectivity structure can be split into distinct modules, wherein each module consists of regions that are more connected to each other than to other regions or modules. In this study, patients with major depressive disorder were randomized to receive either psilocybin or escitalopram and those in the psilocybin arm demonstrated reductions in brain network modularity when comparing pre- vs. post-treatment and the change in brain network modularity was positively associated with treatment outcome (143). These results demonstrated significant changes in within-network connectivity in the DMN and increased inter-network connectivity between the DMN, the EN and the SN. Employing a similar metric in studies of individuals receiving psychedelic therapy with addiction could establish if modularity is a generalized biomarker of psychopathology, and/or a generalized biomarker for treatment response to psychedelic therapy. Further, this metric allows for the assessment of changes in connectivity between key brain network hubs including the DMN, the EN and the SN. In the future longitudinal analysis of changes in RSFC following psychedelic therapy with a consensus-based battery of psychometric tools is advised with larger groups of individuals with addiction and controls.

### Key experiments

a) Acute effects of psychedelics on RSFC of the: DMN, SN and EN in individuals with addiction.
b) Pre- vs. post-psychedelic therapy changes of within and between brain network connectivity (DMN, EN, SN) in patients with addiction as assessed using analysis including dFC, modularity and other standardized RSFC analytical techniques and;
  i) The association of such acute and sub-acute RSFC changes with changes in previously described task-based fMRI techniques as measures of global network functioning during psychological processing may be more sensitive in highlighting latent and more widespread neural disruptions during critical psychological processes in addiction than RSFC analysis alone (144).
  ii) Changes in neurometabolite concentration and its association with changes in cognitive faculties as assessed with behavioral and clinical batteries;
  iii) Changes in neurotransmitter levels as assessed with PET and their association with clinical, behavioral and psychological outcomes associated with addiction
c) Comparison of task-based fMRI with RSFC data in patients treated with psychedelic therapy in addiction to establish which is a more stable and reliable biomarker for neuroplastic and long-term effects.

### Molecular *in vivo* human neuroimaging

*In vivo* molecular neuroimaging in the living human brain has been made possible by the advent of PET and Single Photon Emission Computed Tomography (SPECT). PET measures gamma rays emitted by radioactivity attached to a compound that binds to a known target, for example, a neurotransmitter receptor or protein involved in inflammatory processes or glucose metabolism and is the only direct way to quantify some of the 100 + different neurotransmitters in the living human brain (145). For the last 40 years, neurobiological research in addiction has tried to establish the neurochemical basis of addiction. Analytical approaches have included investigation of changes in brain metabolism, neuroreceptor availability and neurotransmitter release capacity (146). Each of these methods have provided varying levels of detail on the nuances of molecular dysfunction in addiction and hence could be used as a proxy to explore how such parameters may predict response to psychedelic therapies and if psychedelic therapies restore any of the observed molecular deficits. More recently, human molecular neuroimaging has offered insight into the neurochemical mechanism(s) of action of psychedelics although to a much less extent than fMRI (142).

In the following sections we will cover evidence for molecular biomarkers of addiction in-humans and discuss *in vivo* PET imaging techniques to explore the impact of psychedelic therapy on these.

### Neuroreceptor availability

#### Dopamine

Dopamine has been extensively investigated as a biomarker for addiction and dopamine D2/3 receptors (DRD_2/3_) in the striatum have been quantified in several subclasses of addiction using the [^11^C]raclopride radiotracer. Findings from such studies have been mixed, with alcohol and stimulant use disorders appearing to show decreased DRD_2/3_ receptor availability when compared with matched controls. However, no significant between-group differences were found in patients with opiate, cannabis, tobacco and gambling addictions (147) though mood-related impulsivity (‘Urgency’) was negatively correlated with [^11^C]raclopride binding potentials in the GD group (148).

More novel radiotracers such as [^11^C]PHNO have been developed to quantify the relative differences in binding of DRD_3_ compared with dopamine D2 receptor (DRD_2_) in brain regions in order to improve the precision of neuroreceptor identification and gain a deeper understanding of addiction-related biomarkers in alcohol-dependent patients (149) and in stimulant use disorder (150). These findings highlight increased hypothalamus and substantia nigra DRD_3_ in alcohol dependence and stimulant use disorder, respectively, and have led to the development of novel interventions such as the DRD_3_ antagonist.

#### Opioid

The opioid receptor system has also been demonstrated to be upregulated in cocaine users (151), detoxified alcoholic patients (152, 153) and opioid dependence (154) when assessed with the selective mu-opioid receptor (MOR) agonist radioligand [^11^C]carfentanil and is a target of opioid antagonist medication to prevent relapse. More recent data suggest that MOR availability does not differ between pathological gamblers and healthy controls though impulsivity correlated with MOR availability in the caudate in the GD group (155) suggesting this may be a potential target for treatment.

#### GABA

There is also evidence of GABAergic dysregulation in substance and behavioral addictions and in impulsivity. The [^11^C]Ro15-4513 PET radiotracer, selective for the α5 subtype of the benzodiazepine receptor found lower levels of limbic and NAc binding in alcohol and in heroin addiction (156, 157) and higher binding in participants with a history of tobacco smoking (158). Further, in GD there was higher binding in the right hippocampus and amygdala which was positively associated with higher levels of ‘negative urgency’ (159).

These techniques to investigate neuroreceptor differences in addiction have identified several neuroreceptor biomarkers specific to sub-classes of addiction that have been replicated across studies; dopamine receptors in alcohol and stimulant use disorder, opioid receptors in cocaine and opioid dependence and GABA_A_ in alcohol, heroin, smoking and GD when compared with matched controls. The advent of novel radiotracers has allowed for greater specificity in identifying the relative contribution of specific receptor subclasses to addiction neuropathology and these findings have been translated to drug development highlighting the utility of PET imaging in identifying viable drug targets, and hence an area of experimental medicine that should be of great interest for the nascent psychedelic drug development industry. There have been some intriguing associations between facets of impulsivity and receptor dysregulation have been demonstrated across neurotransmitter systems and addiction subclasses demonstrating another targetable avenue for translational psychopharmacology research. To date, there has been no investigation of the effect of psychedelic therapy on neuroreceptor biomarkers in human addiction research though some preclinical work is now establishing psilocybin-induced changes in molecular receptor availability and its association to behavioral assays of alcohol consumption and treatment outcome in animal models of alcohol addiction (160). Thus, evidence for psilocybin restoring molecular dysfunction in addiction now needs to be translated to human research, given the ample opportunity presented by the identified neuroreceptor biomarkers described, the basic neuromolecular mechanisms of action leading to therapeutic efficacy needs to be established.

### Neurotransmitter release capacity

Addiction has been proposed as a ‘reward deficient’ state, which is compensated for with substance use (92). Several radioligands have been developed to assess theories of deficits in neurotransmission and in particular assessment of blunted dopamine, endorphins and serotonin can help to establish the extent of molecular dysfunction in addiction and to see whether these pathological hallmarks are therapeutically modulated by psychedelics. The method to assess neurotransmission in the human brain is through conducting neurotransmitter release studies. These rely on the principle that psychostimulants, such as methylphenidate or amphetamines, can reliably increase levels of endogenous dopamine (161), opioids (162) and more recently serotonin (163). Non-pharmacological manipulations include stress, motor tasks, video games, and cue-induced craving (164).

### Dopamine release

There is evidence for blunted dopamine release in cocaine, opiate, and alcohol dependence but not cannabis dependence as assessed with the antagonist radioligand [11]raclopride (147). A recent review demonstrated that, behaviorally, blunted striatal dopamine transmission could reflect increased impulsivity and altered cost/benefit computations that are associated with addiction. The data suggest that blunted dopamine neurotransmission is a biomarker for increased risk of developing addiction and treatment-resistance, which is associated with impulsive behavior and reduced motivation and is linked to increased drug consumption (165). Theoretically, if psychedelics lead to clinically effective outcomes then this should be reflected in increases in endogenous dopamine neurotransmitter levels in addiction disorders which are reflected by improved brain function in addiction processed and associated normalization of neurotransmitter deficit. Intriguingly some studies have found that 5-HT_2A_R agonism induces dopamine release and that 5-HT_2A_R co-activation with dopamine is necessary to mediate activation of the brain’s reward circuitry (166) which suggests a plausible pharmacologically relevant activation cascade.

To directly test the effects of psychedelic therapy on neurotransmission and brain function in humans with addiction requires advanced multimodal neuroimaging techniques. Several novel agonist radiotracers including [^11^C]PHNO have been found to be sensitive to endogenous dopamine (167). Utilizing this radiotracer in a multimodal fMRI-PET study with behaviorally salient, dopamine-enhancing tasks such as monetary reward paradigms, that probe addiction-related brain processes, could theoretically release dopamine in the living human brain. The extent to which psychedelic therapy remediates the observable neurotransmitter and functional deficits and their relationship to clinical outcomes would offer unparalleled insight into this intervention. This would provide evidence for the ‘molecular-functional-clinical’ translational explanatory bridge, which so far in psychiatric psychopharmacology research has not been conducted, and would provide the most advanced biopsychosocial theory of psychedelics in treating addiction.

### Endorphin release

Recent research has highlighted the importance of endogenous opioids in addiction. B-endorphin, which is one the brain’s natural endorphins. Has been shown to bind at the MOR mediating euphoric and analgesic effects (168) and can be released in the living human brain by oral dexamphetamine (162). Addiction has been conceptualized as a multiple-neurotransmitter disorder with evidence of blunted dexamphetamine-induced endorphin release in patients with alcohol (169) and pathological gambling as assessed with [^11^C]carfentanil when compared with healthy controls (170). This study also showed reduced amphetamine-induced euphoria and alertness. These findings are consistent with growing evidence that dysregulation in opiate tone is consistent across both substance use and behavioral addictions, in the absence of changes in receptor availability and thus may play an important role in the pathophysiology of addictions broadly. Hence, this could be used as a surrogate addiction molecular biomarker to assess the effects of psychedelic therapy.

### Serotonin release

[^11^C]Cimbi-36 is the first agonist radioligand suitable for the examination of cortical 5-HT_2A_R (171). Recent work has demonstrated this radiotracer to be sensitive to endogenous serotonin following administration of oral dexamphetamine in healthy humans (163) and in clinical populations including depression demonstrating reductions in serotonin release capacity in the latter when compared with healthy controls (172). As yet, these novel PET ligands have not been widely employed in translational psychopharmacology research and not at all in addiction. The opportunity presented by the development of such radioligands presents for the first time the ability to assess cortical serotonergic molecular biomarkers in addiction and the impact of psychedelic therapies on modulating any observable deficits.

### Key experiments

a) PET imaging of dopamine, opioid and GABA receptor availability pre – vs. post psychedelic therapy in addiction populations to assess changes in neuroreceptor availability and its association with clinical outcome and facets of impulsivity (selection of neuroreceptor to investigate must be dependent on evidence for dysfunction in the specific disorder which has been described)
b) Multimodal FMRI-PET imaging to assess changes in dopaminergic modulation in relation to monetary reward tasks in addiction populations pre- vs. post-psychedelic therapy. This would be the first ‘molecular-functional-clinical’ mechanistic pathway for its role in addiction. These results would allow for the identification of molecular and functional stratification and prognostic biomarkers and would greatly advanced the field of psychedelic addiction drug development. Such multimodal imaging platforms are advised in all fields of psychiatric and neurological drug development and represent the most advanced area of experimental translational neuroscience research
c) PET neurotransmitter release studies of novel dopamine, opioids and serotonin radiotracers to assess the changes in neurotransmitter tone following psychedelic therapy. There is evidence that dysregulation in neurotransmitter tone may be a more pleiotropic biomarker of addiction due to its presence in addiction populations in the absence on neuroreceptor abnormalities. Selection of radiotracer should be dependent on the addiction sub-population being investigated and based on previous evidence of dysregulation in the specific system.

### Molecular neuroplasticity

Neuroplasticity is emerging as a novel and possible convergent neurotherapeutic mechanism of action for classic and non-classic psychedelics. This mechanism is theorized to be effective across a range of psychopathologies characterized by ‘canalisation’ [for a thorough review please see: (173, 174)]. In brief, the assessment of neuroplasticity can focus on changes at a cellular and molecular level using PET ligands focusing on several proteins including; synaptic glycoprotein 2A (SV2A, a marker of pre243 synaptic terminals; [^11^C] UCB-J) and mitochondrial complex 1 (MC1, a marker of mitochondrial density; [^18^F]BCPP-EF) offer promising prospects (142). These techniques could investigate pathological biomarkers of plasticity with evidence for reduced SV2A in the PFC in CUD (175). This marker was sensitive to the frequency of recent cocaine use and negatively correlated with abstinence and hence could be a plausible biomarker to predict treatment response to interventions such as psychedelic therapy. Interestingly, another study recently found SV2A to be upregulated in the pig brain 1 and 7 days after the pig was given a single psychedelic dose of psilocybin (176) which suggests a plausible restorative effect of psilocybin on pathological frontal plasticity. However, so far, there are no data available from studies in humans who have taken psychedelic drugs and none in clinical populations with addiction. These molecular approaches can be integrated with electroencephalography (EEG) techniques that assess brain neuroplasticity such as the visual long-term potentiation (VLTP) and mismatched negativity paradigms (MMN) (177).

## Conclusion

Addiction suffers the highest levels of unmet medical needs of all mental health conditions (178), with the current armamentarium providing modest impact on patients’ lives and failing to address remarkably high rates of treatment resistance, relapse and mortality (179). In this review, we have summarized the past, present, and future of research investigating psychedelic therapies for addiction. Approaching nearly a century since its introduction into Western addiction medicine, psychedelic therapy has demonstrated clinical success across a range of settings from the real world to controlled clinical research, and more recently double-blind randomized controlled clinical trials. Therapeutic effects have been observed across classic and non-classic psychedelics and with the advent of larger phase III clinical trials, it is highly plausible that these medicines will receive regulatory licensing for patients within this decade. Despite these promising clinical signals, there has been a dearth of research exploring the biological and psychological factors that mediate treatment outcomes. We argue that biomedical and neuropsychopharmacological techniques that have traditionally been used in addiction research over the last 40 years should now be redeployed to the study of psychedelic therapies adjunctive to clinical trials in humans with addiction disorders. These techniques have enabled a deeper understanding of the neuropathology of addiction and can be used to examine the neurotherapeutic application of psychedelic therapy in the context of addiction biomarkers covering functional, molecular and structural deficits. Such an approach also enables for biomarker informed prognosis, ultimately to enable precision-based stratification of patients to specific treatments with the ultimate goal of enabling a personalized medicine approach that will ultimately improve patient outcomes.

## Author contributions

RZ wrote and developed the initial manuscript. RH and MW provided feedback on fMRI. TB provided literature on real-world evidence. MS, SS, CA, and LR provided proofing and comments on overall the structure. DN and DE had overview of the work and provided overall guidance. All authors contributed to the article and approved the submitted version.

## Conflict of interest

MW was employed by Invicro.

The remaining authors declare that the research was conducted in the absence of any commercial or financial relationships that could be construed as a potential conflict of interest.

## Publisher’s note

All claims expressed in this article are solely those of the authors and do not necessarily represent those of their affiliated organizations, or those of the publisher, the editors and the reviewers. Any product that may be evaluated in this article, or claim that may be made by its manufacturer, is not guaranteed or endorsed by the publisher.
